# Supplementary material for: Molecular mechanisms of re-emerging chloramphenicol susceptibility in extended-spectrum beta-lactamase-producing Enterobacterales
Source: Nat Commun. 2024 Oct 18;15:9019. doi: 10.1038/s41467-024-53391-2 (PMC11489765; doi:10.1038/s41467-024-53391-2)
Supplement: Supplementary file 1 — Supplementary information [file 41467_2024_53391_MOESM1_ESM.pdf]

Molecular mechanisms of re-emerging chloramphenicol susceptibility in extended-spectrum beta-lactamase producing Enterobacterales

Supplementary information:

Supplementary Figure 1 - Heatmap of phenotypic chloramphenicol resistance

Supplementary Figure 2 – dCAT assay

Supplementary Figure 3 – Assemblies and percentage identity of *catB4*.

Supplementary Figure 4 – PCR probing of *catA1*

Supplementary Figure 5 – HRM assay

Supplementary Figure 6 – Stability of IS*5-catA1* upon CHL selection

Supplementary Figure 7 – Co-occurrence networks of AMR genes

Supplementary Figure 8 – Frequency distribution of *catB3*

Supplementary Figure 9 – Number of isolates per sequence type

Supplementary Table 1 – Primers used in this study

Supplementary Table 2 – Long-read stats

Supplementary methods

Supplementary references

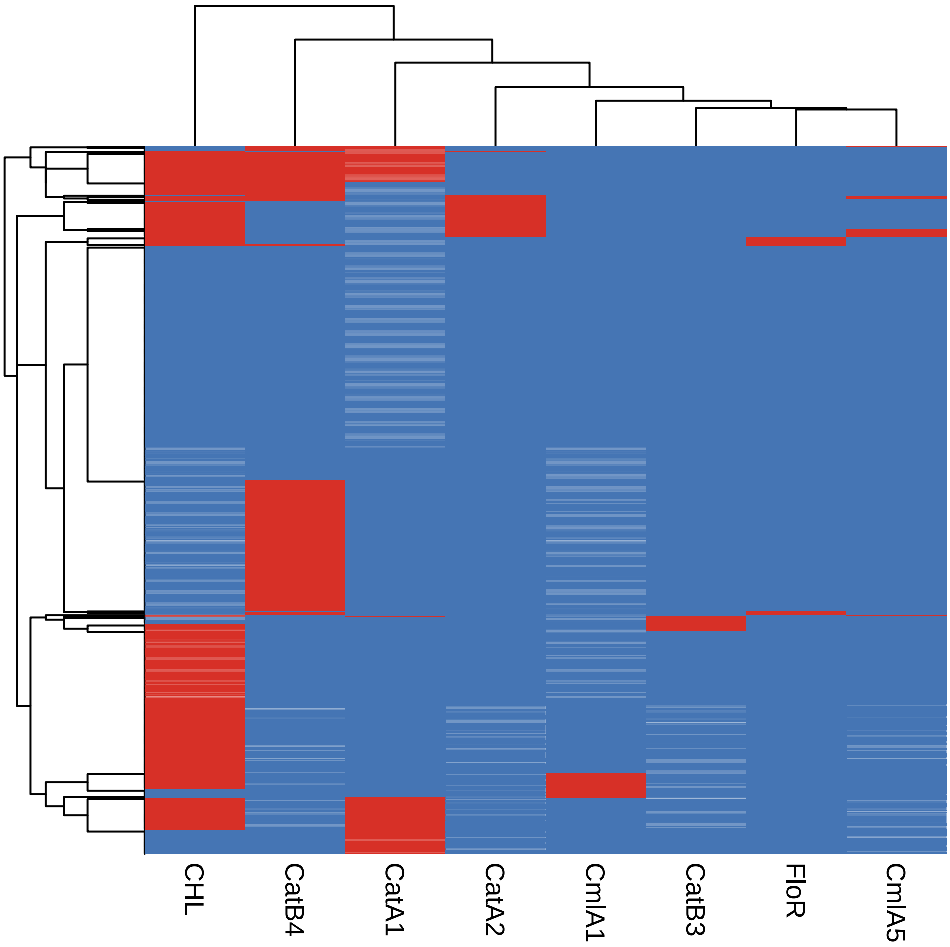

Supplementary Figure 1 | Heatmap of phenotypic chloramphenicol resistance and identified resistance genes for chloramphenicol. Each row represents one isolate; red indicates presence; blue absence. Heatmap is row and column clustered. Source data are provided as a Source Data file.

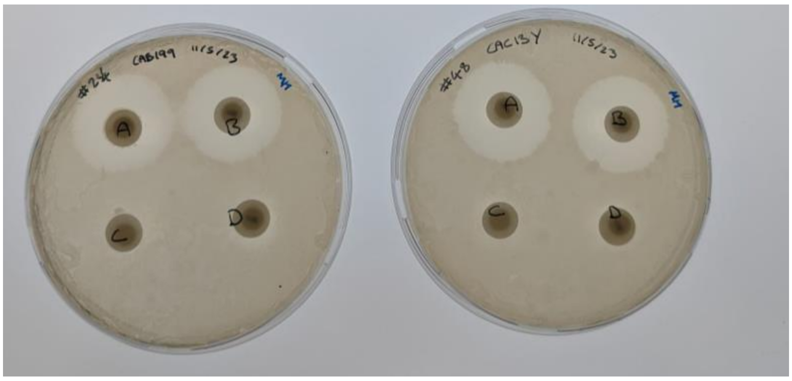

Supplementary Figure 2 | dCAT assay measuring cross-protection from CAT enzyme producing isolates. Labels on plate: A tested isolate, B susceptible control, C susceptible - control without chloramphenicol (CHL) disc, D + control (*catA1*), Isolates CAB119 & CAC13Y fail to inactivate CHL from disc.

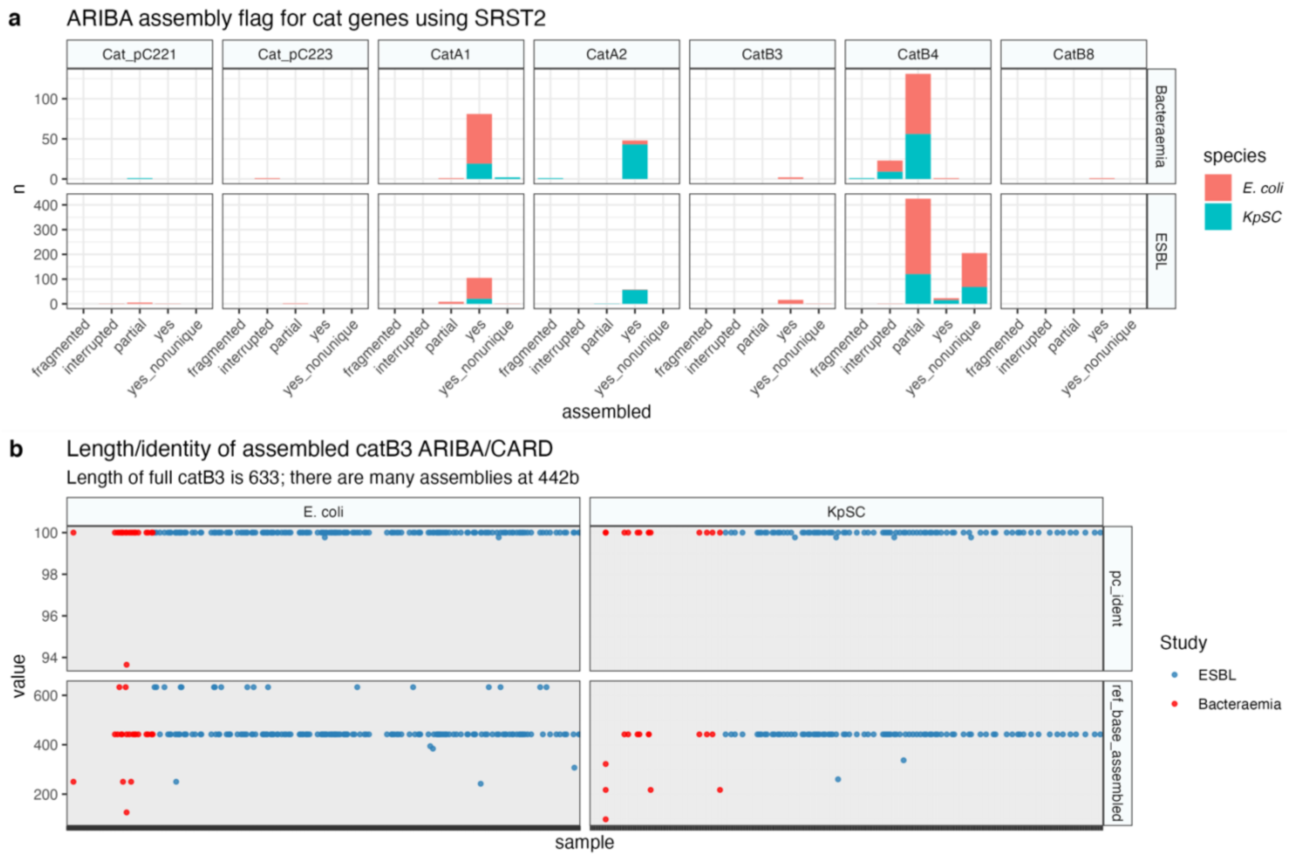

Supplementary Figure 3 | Assemblies and percentage identity of *catB4* a assemblies of *cat* genes among the ESBL and the bacteraemia sentinel isolates using Ariba/SRST2. Fully assembled (yes), fully assembled multiple times (yes_nonunique), partially assembled (partial) or interrupted assembly (interrupted). **b** Percentage identity and number of bases assembled to ref of *catB4* compared to *catB3* from the CARD database. Source data are provided as a Source Data file.

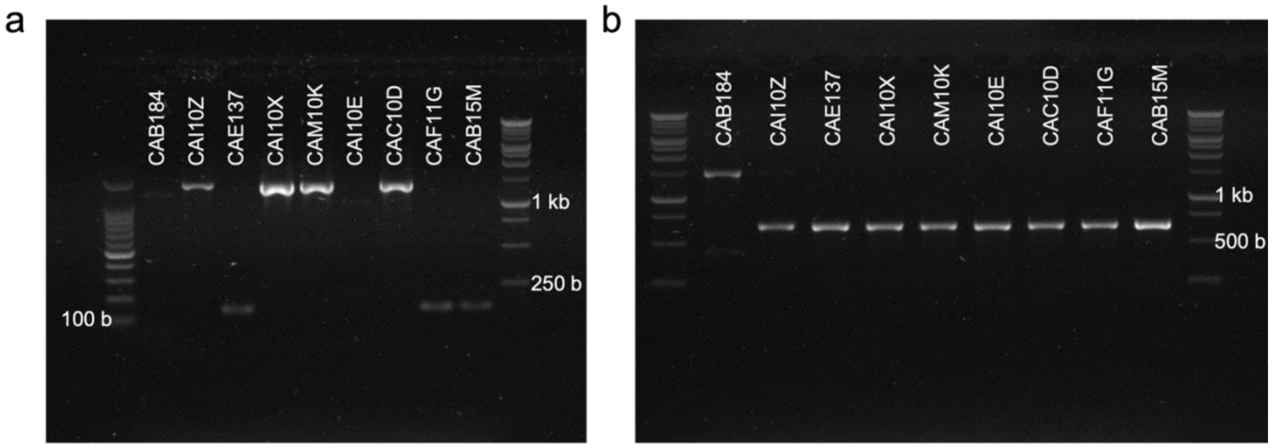

Supplementary Figure 4 | PCR probing for *catA1*. PCR amplicon ran on 1% agarose gel: **a** *catA1* probing: expected size 150 bp. Lane 1: 100 b Marker, lane 11: 1kb plus Marker (Promega, UK). **b** catA1-CDS primers: expected amplicon size 660 bp. Lane 1 & 11: 1kb plus Marker (Promega, UK).

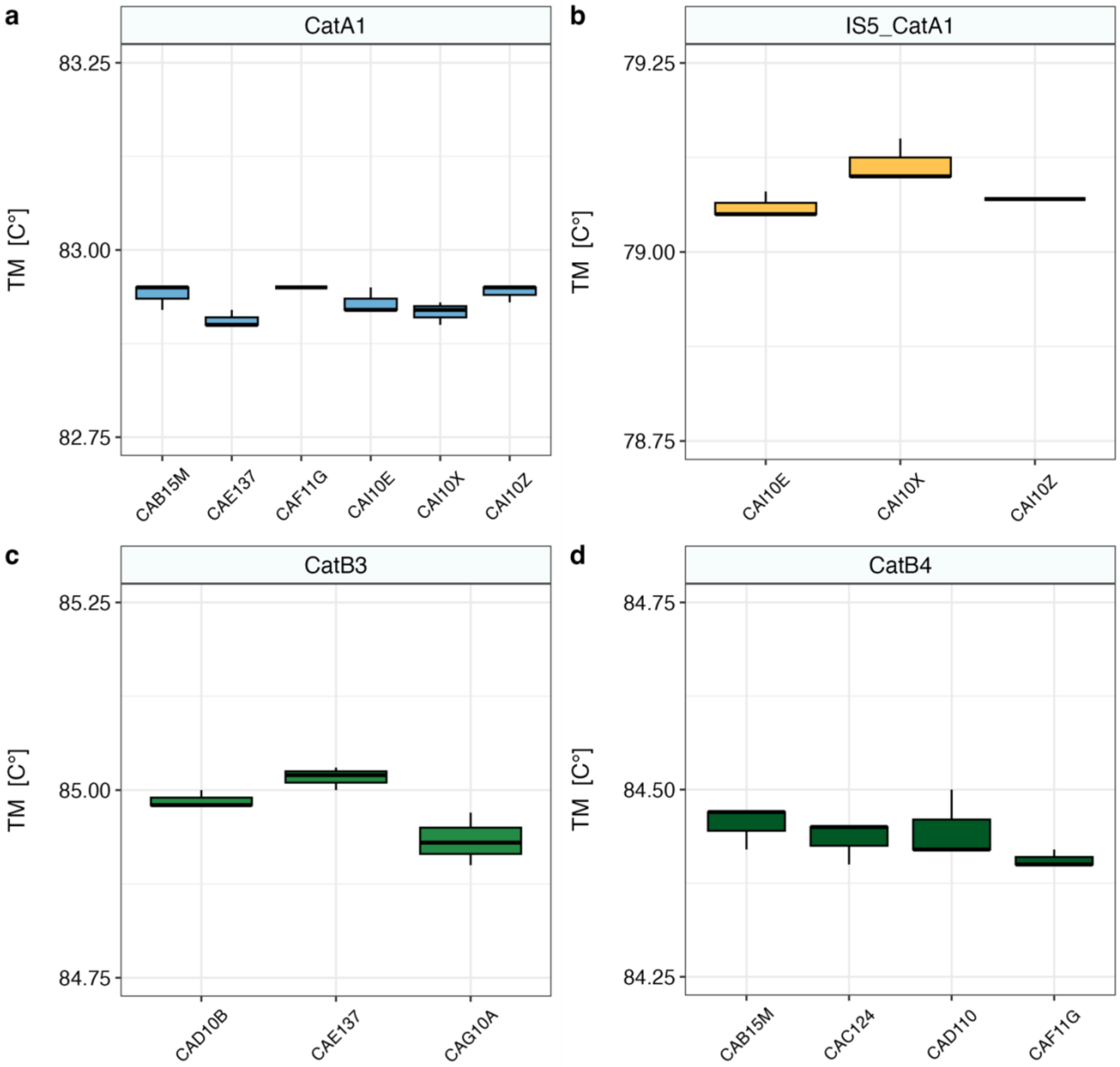

Supplementary Figure 5 | High-resolution melting (HRM) assay probing for **a** *catA1*, **b** IS*5_catA1*, **c** *catB3* and **d** *catB4* (= *catB3*$\Delta^{443\text{-}633}$). TM = melting temperature. Each boxplot represents a single isolate with 3 technical replicates. Source data are provided as a Source Data file.

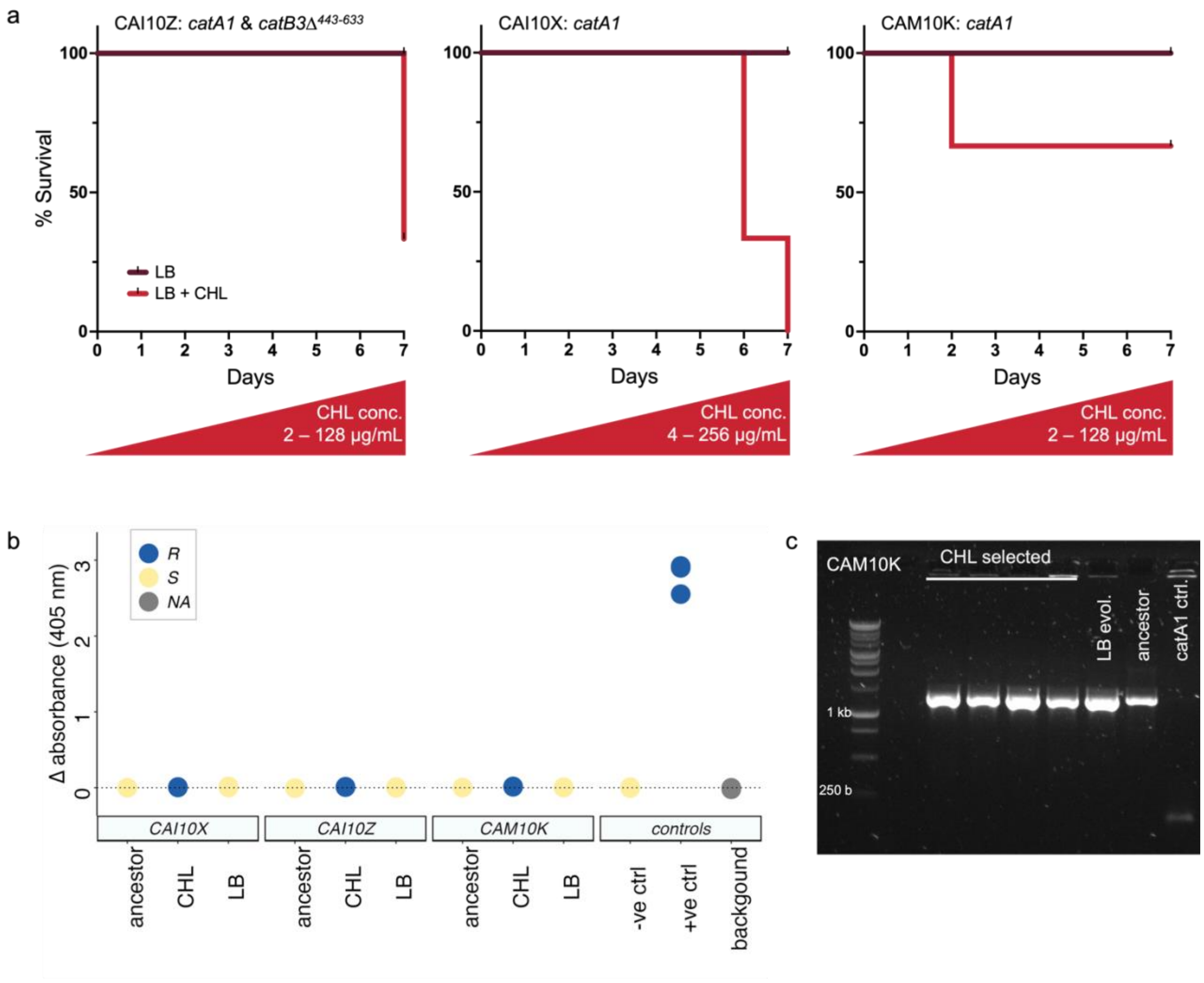

Supplementary Figure 6 | Stability of IS*5-catA1* upon CHL selection. a Survival curves of experimentally evolved populations in LB (dark red) and with increasing concentrations of CHL (red) for isolates CAI10X, CAI10Z and CAM10K (all n=3 for both LB & CHL). **b** rCAT assay performed with last surviving replicate populations of CHL- and LB selected and ancestor population for each isolate. Source data are provided as a Source Data file. **c** PCR probing for *catA1:* Colony PCR from isolate CAM10K. Lane 1 1kb plus Marker (Promega, UK), lane 2 empty, lanes 3-6 four colonies from 2 independently evolved populations with increasing CHL concentrations from Day 7. Lane 7 LB evolved control population, lane 8 ancestor clone and lane 9 *catA1* wild-type control isolate.

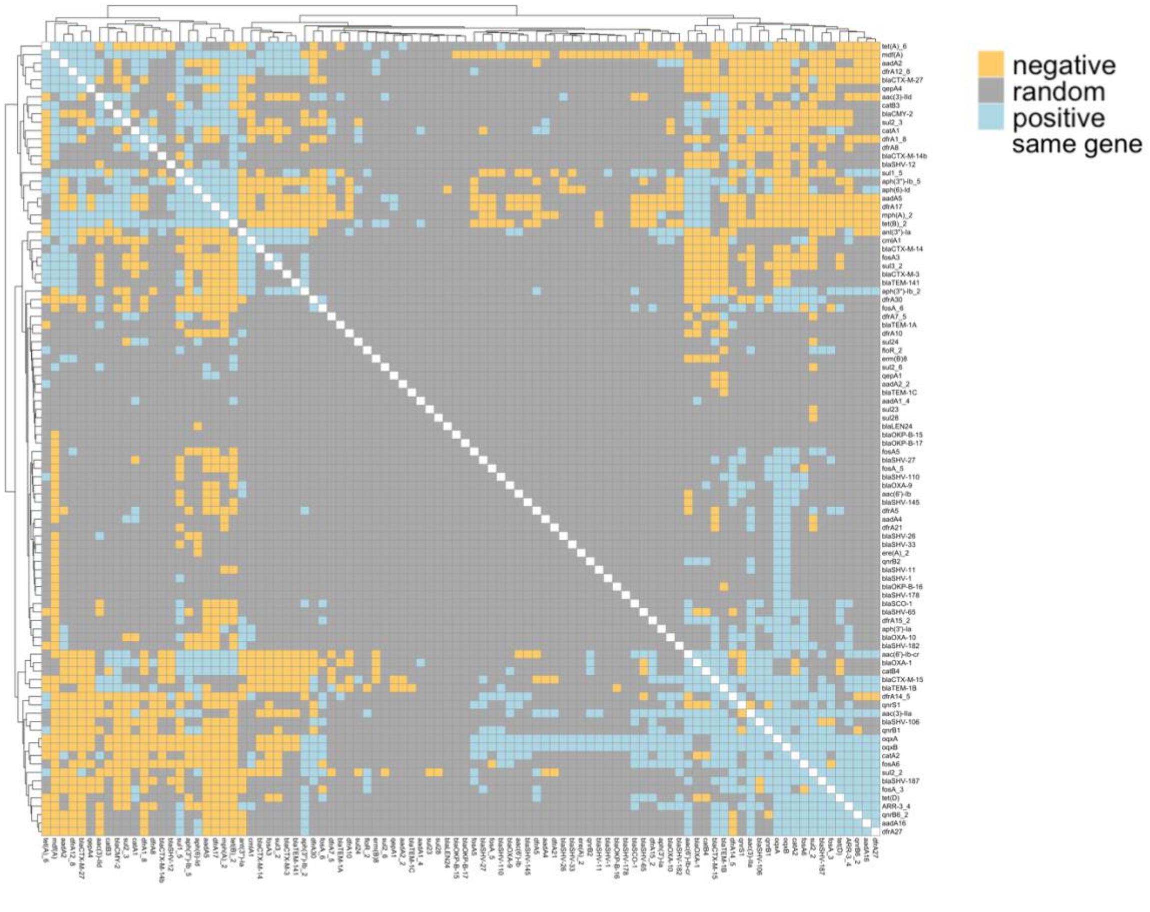

Supplementary Figure 7 | Co-occurrence networks of AMR genes. Heatmap displaying co-occurrence relationships between AMR genes as either positive (blue), random (grey) or negative (orange). These are probabilistic values based on the difference in expected and observed frequencies of co-occurrence between each pair of genes, these values were obtained by applying the probabilistic model from [1]. Co-occurrence across all AMR genes clustering using Euclidian distance and the complete method. Source data are provided as a Source Data file.

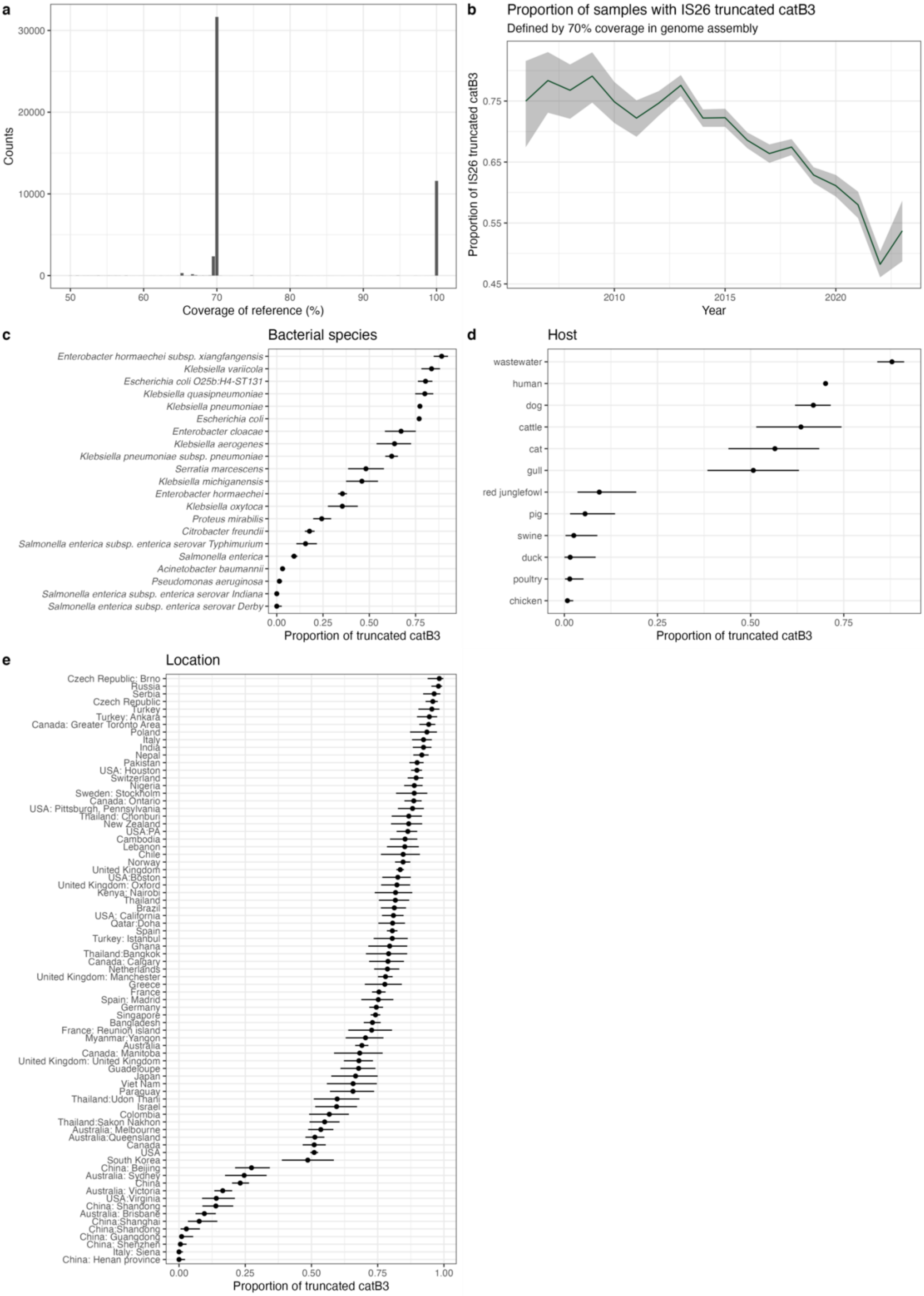

a
Counts
Coverage of reference (%)
b
Proportion of samples with IS26 truncated catB3
Defined by 70% coverage in genome assembly
Proportion of IS26 truncated catB3
Year
c
Bacterial species
Enterobacter hormaechei subsp. xiangfangensis
Klebsiella variicola
Escherichia coli O25b:H4-ST131
Klebsiella quasipneumoniae
Klebsiella pneumoniae
Escherichia coli
Enterobacter cloacae
Klebsiella aerogenes
Klebsiella pneumoniae subsp. pneumoniae
Serratia marcescens
Klebsiella michiganensis
Enterobacter hormaechei
Klebsiella oxytoca
Proteus mirabilis
Citrobacter freundii
Salmonella enterica subsp. enterica serovar Typhimurium
Salmonella enterica
Acinetobacter baumannii
Pseudomonas aeruginosa
Salmonella enterica subsp. enterica serovar Indiana
Salmonella enterica subsp. enterica serovar Derby
Proportion of truncated catB3
d
Host
wastewater
human
dog
cattle
cat
gull
red junglefowl
pig
swine
duck
poultry
chicken
Proportion of truncated catB3
e
Location
Czech Republic: Brno
Russia
Serbia
Czech Republic
Turkey
Turkey: Ankara
Canada: Greater Toronto Area
Poland
Italy
India
Nepal
Pakistan
USA: Houston
Switzerland
Nigeria
Sweden: Stockholm
Canada: Ontario
USA: Pittsburgh, Pennsylvania
Thailand: Chonburi
New Zealand
USA:PA
Cambodia
Lebanon
Chile
Norway
United Kingdom
USA:Boston
United Kingdom: Oxford
Kenya: Nairobi
Thailand
Brazil
USA: California
Qatar:Doha
Spain
Turkey: Istanbul
Ghana
Thailand:Bangkok
Canada: Calgary
Netherlands
United Kingdom: Manchester
Greece
France
Spain: Madrid
Germany
Singapore
Bangladesh
France: Reunion island
Myanmar:Yangon
Australia
Canada: Manitoba
United Kingdom: United Kingdom
Guadeloupe
Japan
Viet Nam
Paraguay
Thailand:Udon Thani
Israel
Colombia
Thailand:Sakon Nakhon
Australia: Melbourne
Australia:Queensland
Canada
USA
South Korea
China: Beijing
Australia: Sydney
China
Australia: Victoria
USA:Virginia
China: Shandong
Australia: Brisbane
China:Shanghai
China:Shandong
China: Guangdong
China: Shenzhen
Italy: Siena
China: Henan province
Proportion of truncated catB3

Supplementary Figure 8 | Frequency of *catB3* and truncated *catB3*. a Number of isolates (n = 46,667) with *catB3* according to their coverage (in %) to the reference gene. Data downloaded from MicroBIGG-E (4th August 2023). Proportion of truncated *catB3* and confidence interval (**b**) over time including data with > 100 isolates per year, **(c)** by species (> 100 isolates), **(d)** by host (> 50 isolates) and **(e)** geographic location (> 100 isolates). Confidence intervals, where shown, are exact binomial confidence intervals. Source data are provided as a Source Data file.

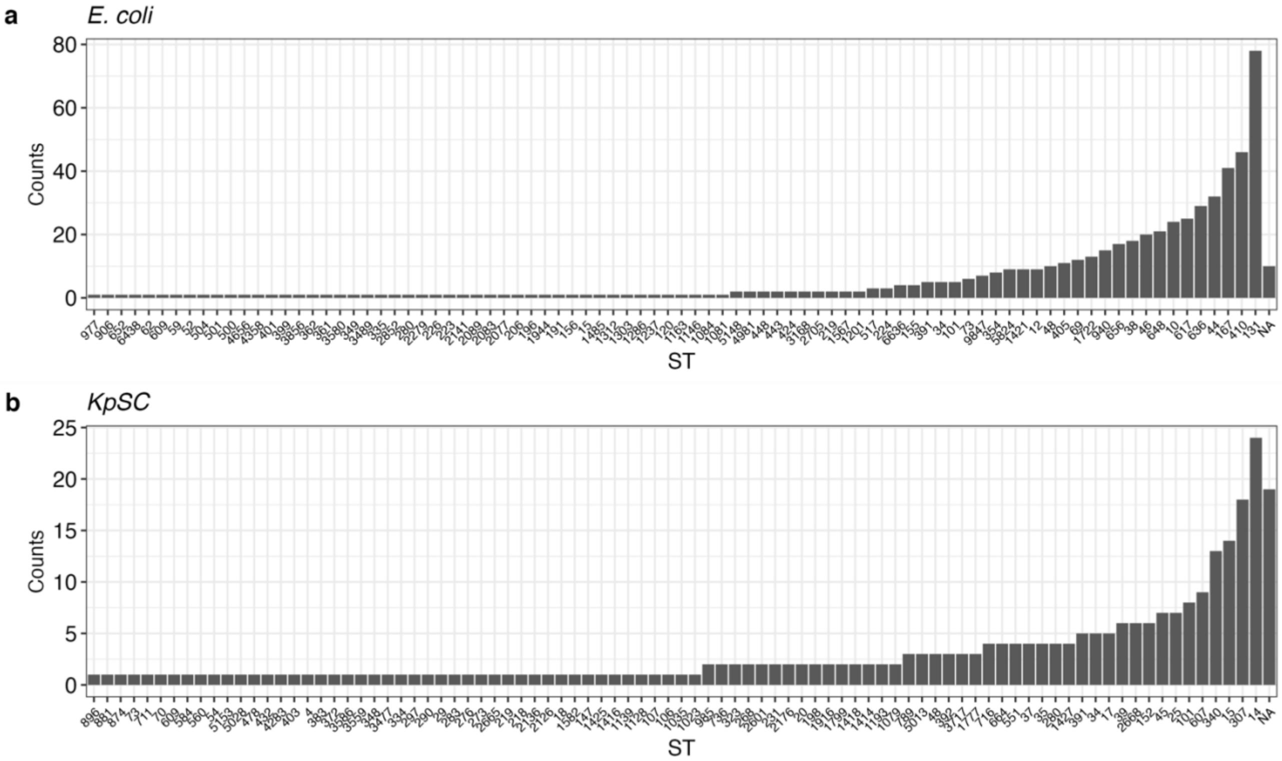

Supplementary Figure 9: Number of isolates per sequence type for *E. coli* (a) and *KpSC* (b) of the 840 Malawian genomes included in this study. NA = not determined or novel ST. Source data are provided as a Source Data file.

Supplementary Table 1 – Primers used in this study

	Forward Primer (5'-3')	Reverse Primer (5'-3')	Reference
Cloning primers			
catA1-CDS	tttaagaaggagatatacatATGGAGAAAAAAATCACTGGAT	cctgcaggtctggacatttaTTACGCCCCGCCCTGCC	This study
catA2-CDS	tttaagaaggagatatacatATGAATTTTACCCGGATTGACC	cctgcaggtctggacatttaTTATTTCAGTATGTTATCACAC	This study
catB3-CDS	tttaagaaggagatatacatATGACCAACTACTTTGATAGCC	cctgcaggtctggacatttaTTAGACGGCAAACTCGAGC	This study
catB4-CDS	tttaagaaggagatatacatATGACCAACTACTTTGATAGCC	cctgcaggtctggacatttaTTATGGTGATCCCCTGG	This study
pEB1-backbone	TAAATGTCCAGACCTGCAGG	ATGTATATCTCCTTCTTAAATCTAG	This study
pEB1_sequencing	GCGTATCACGAGGCCCTTTC	AAAGGGAAAACTGTCCATATGCAC	This study
Probing primers			
catA1	AATAAGATCACTACCGGGCGT	GCAACTGACTGAAATGCCTCA	[2]
HRM primers			
catA1	TTTCGTCTCAGCCAATCCCT	CGACATGGAAGCCATCACAA	This study
IS5-catA1	CAGGAACTTGTTCGCACCTT	GGCCGGATAAAACTTGTGCT	This study
catB3	GAAACGCTTCACCGATGAGG	GCCGCTTTGATCTTCTCCAG	This study
catB4	CAATGACGTTTGGATCGGCT	CAACAGTGCCCCACATCTTT	This study

Lower case = overhangs homologous with pEB1-backbone. NB: Forward cloning primer for *catB3* and *catB4* is identical.

Supplementary Table 2 – Long-read assembly statistics, qc and accession numbers.

ID	Biosample accession number	CHL R/S	CHL-R genes	number of contigs	sum length of all contigs	minimum length contig	average length of contigs	max length contig	Q1	Q2	Q3	N50
CAE137	SAMN38266307	R	catB3, catA1	6	5,516,907	3,204	919,484.50	5,334,490	12,238	17,293.50	132,388	5,334,490
CAD110	SAMN38266308	R	catA2, catB3$\Delta^{443\text{-}633}$	4	5,019,608	3,588	1,254,902	4,867,646	10,195	74,187	2,499,609	4,867,646
CAD10B	SAMN38266309	R	catB3	13	5,040,963	2,972	387,766.40	4,802,091	5,893	15,383	24,306	4,802,091
CAC124	SAMN38266310	S	catB3$\Delta^{443\text{-}633}$	6	5,584,617	16,028	930,769.50	5,315,926	16,872	29,146.50	177,498	5,315,926
CAC10A	SAMN38266311	R	catA1	2	4,867,656	125,633	2,433,828	4,742,023	125,633	2,433,828	4,742,023	4,742,023

Supplementary methods

Functional CAT assay (dCAT)

The disk-diffusion CAT (dCAT) assay was adapted from [3]. Cultures of CHL a sensitive *E. coli* isolate (EC1010805 from [4]) was prepared in MH2 broth and grown overnight at 37°C, 220 rpm. Isolates to be tested and positive and negative control isolates (KI D49363 from [5] and EC1010805) were streaked onto Muller Hinton agar (MH 70191, Merck) and incubated overnight at 37°C. Overnight culture of EC1010805 was diluted 1:10 in MH2 broth and 500 μl spread onto MH agar plates and left to dry for 1 hour at RT. Four discs of 10mm Grade 1 filter paper (1001-6508, Whatman) were added to four points of the agar plate labelled A-D. These related to: A) isolate to be tested with CHL; B) negative control with CHL; C) negative control without CHL; D) positive control with CHL. Using a 1μl loop, a small mass of the relevant bacterial isolate from the MH plate was spread over the filter paper. After a 5-minute incubation at RT, a 30 μg/ml chloramphenicol disc (CT0013B, Oxoid) was placed onto positions A, B and D and a blank disc (CT0998B, Oxoid) was placed onto position C. Plates were incubated overnight at 37°C. QC: Position B should have a zone of inhibition, positions C and D should have no (or a small zone for D) zone of inhibition. If these conditions are met and the isolate of interest (A) does not have a zone of inhibition this indicates CAT activity. If QC checks fail, assay should be repeated.

High resolution melting assay

Each HRM assay was performed using 6.25 µL of 2x Type-it HRM PCR buffer (Qiagen, Germany), all primers were added to a final concentration of 400 nM. Molecular grade water was then added to a final reaction volume of 12.5 µl, including 2.5 µl of DNA template. Reactions were thermally cycled in a RGQ 6000 (Qiagen), with the following thermal profile: Taq activation at 95 °C for 5 minutes, followed by 25 cycles of 95 °C for 10 seconds, 58 °C for 30 seconds, 72 °C for 15 seconds. Following this, HRM was carried out by melting from 74 °C to 88 °C, taking a reading in the HRM channel every 0.1 °C, with a 2 second stabilisation between each step. Data was visualised as the negative first derivative of the melting curve to show peak fluorescence dissociation and the predictive TM of the

target was recorded. All analysis was carried out using the RGQ system software. All samples were assayed in triplicate.

Supplementary references

1. Veech JA, Peres-Neto P. A probabilistic model for analysing species co-occurrence. *Global Ecology and Biogeography* **22**, 252-260 (2013).

2. Williams CT, Musicha P, Feasey NA, Adams ER, Edwards T. ChloS-HRM, a novel assay to identify chloramphenicol-susceptible Escherichia coli and Klebsiella pneumoniae in Malawi. *J Antimicrob Chemother* **74**, 1212-1217 (2019).

3. Slack MP, Wheldon DB, Turk DC. Rapid detection of chloramphenicol resistance in Haemophilus influenzae. *Lancet* **2**, 1366 (1977).

4. Musicha P*, et al.* Genomic landscape of extended-spectrum beta-lactamase resistance in Escherichia coli from an urban African setting. *J Antimicrob Chemother* **72**, 1602-1609 (2017).

5. Musicha P*, et al.* Genomic analysis of Klebsiella pneumoniae isolates from Malawi reveals acquisition of multiple ESBL determinants across diverse lineages. *J Antimicrob Chemother* **74**, 1223-1232 (2019).

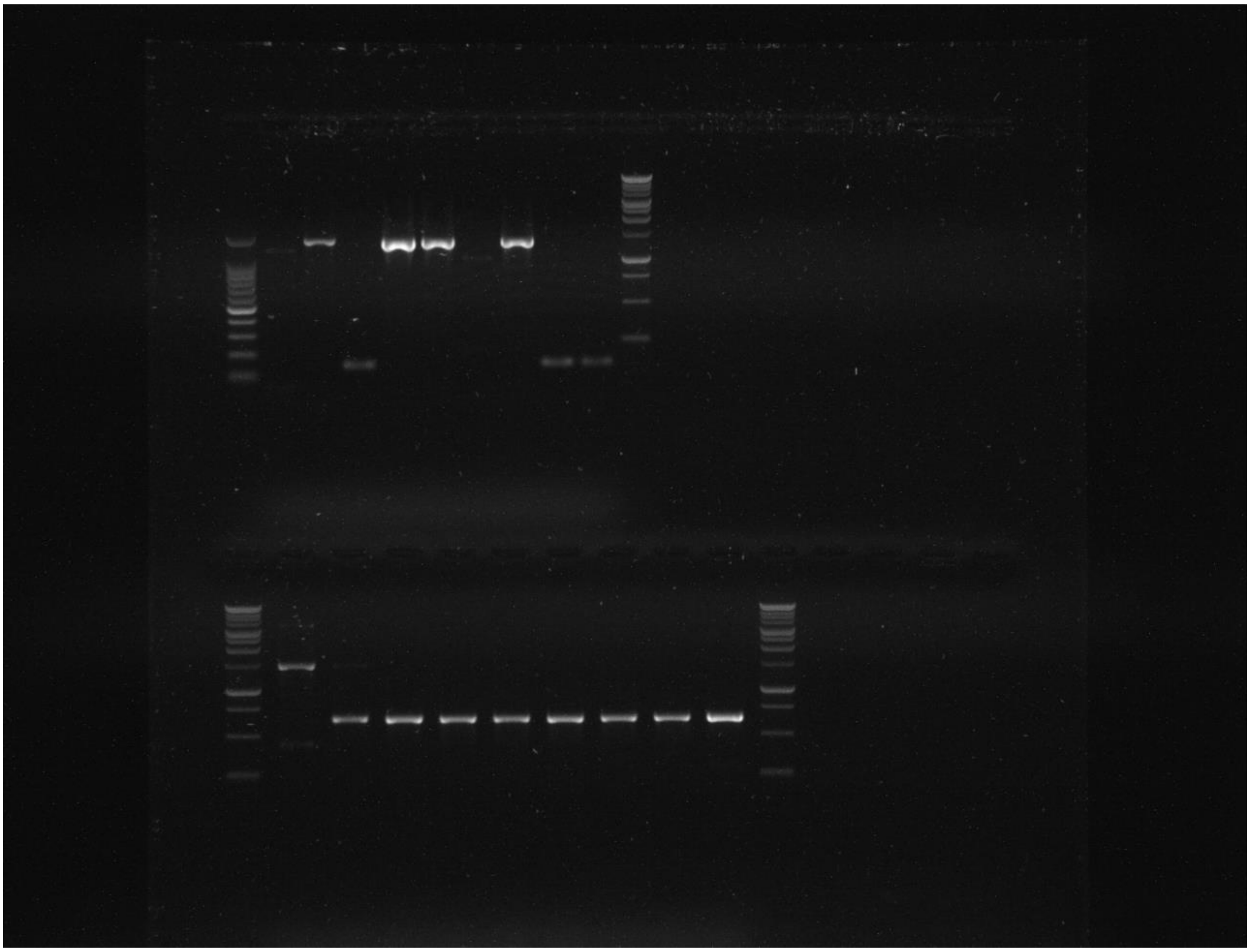

Uncropped Gel image for Supplementary Figure 4a (top) and b (bottom).

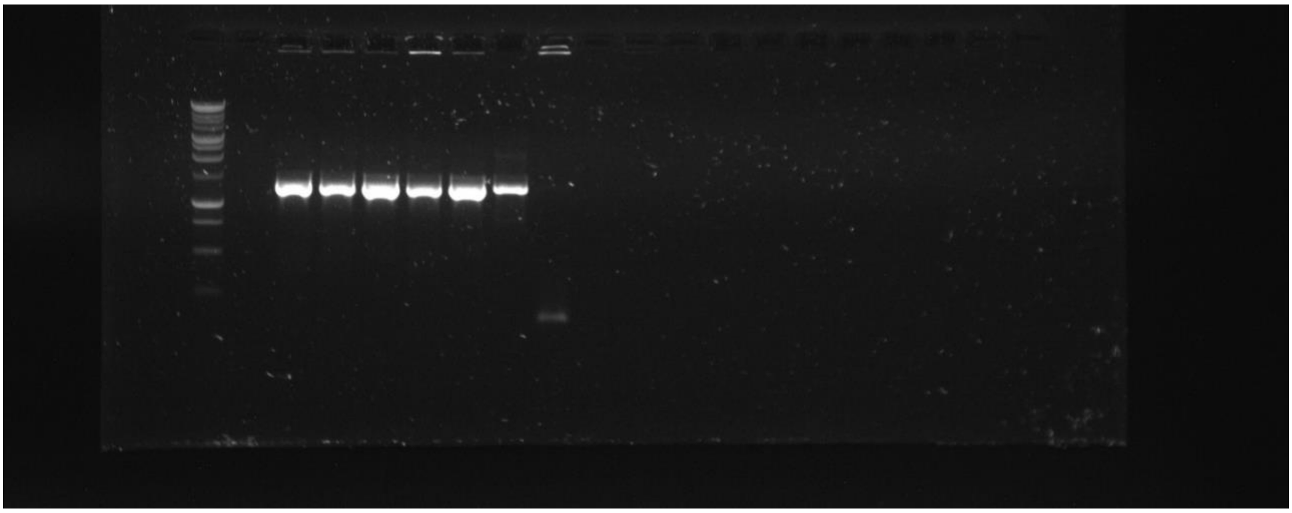

Uncropped Gel image for Supplementary Figure 6c).
