# Supplementary material for: Molecular mechanisms of re-emerging chloramphenicol susceptibility in extended-spectrum beta-lactamase-producing Enterobacterales
Source: Nat Commun. 2024 Oct 18;15:9019. doi: 10.1038/s41467-024-53391-2 (PMC11489765; doi:10.1038/s41467-024-53391-2)
Supplement: Supplementary file 3 — Description of additional supplementary files [file 41467_2024_53391_MOESM3_ESM.pdf]

<u>Description of Additional Supplementary Files</u>

Supplementary Data 1: Strain ID, sequence type (ST), Species, chloramphenicol susceptibility, Presence/Absence (1/0) of CatA1, CatA2, CatB4, CatB3, FloR, CmlA1, CmlA5 genes, study/source of isolate, and ENA accession.

Supplementary Data 2: Co-occurrence matrix
