# Supplementary material for: Molecular mechanisms of re-emerging chloramphenicol susceptibility in extended-spectrum beta-lactamase-producing Enterobacterales
Source: Nat Commun. 2024 Oct 18;15:9019. doi: 10.1038/s41467-024-53391-2 (PMC11489765; doi:10.1038/s41467-024-53391-2)
Supplement: Supplementary file 6 — Reporting Summary [file 41467_2024_53391_MOESM6_ESM.pdf]

Corresponding author(s): Fabrice Graf

Last updated by author(s): Oct 1, 2024

Reporting Summary

Nature Portfolio wishes to improve the reproducibility of the work that we publish. This form provides structure for consistency and transparency in reporting. For further information on Nature Portfolio policies, see our Editorial Policies and the Editorial Policy Checklist.

Statistics

For all statistical analyses, confirm that the following items are present in the figure legend, table legend, main text, or Methods section.

n/a	Confirmed	
☐	☒	The exact sample size (n) for each experimental group/condition, given as a discrete number and unit of measurement
☐	☒	A statement on whether measurements were taken from distinct samples or whether the same sample was measured repeatedly
☐	☒	The statistical test(s) used AND whether they are one- or two-sided<br>*Only common tests should be described solely by name; describe more complex techniques in the Methods section.*
☒	☐	A description of all covariates tested
☒	☐	A description of any assumptions or corrections, such as tests of normality and adjustment for multiple comparisons
☐	☒	A full description of the statistical parameters including central tendency (e.g. means) or other basic estimates (e.g. regression coefficient) AND variation (e.g. standard deviation) or associated estimates of uncertainty (e.g. confidence intervals)
☒	☐	For null hypothesis testing, the test statistic (e.g. F, t, r) with confidence intervals, effect sizes, degrees of freedom and P value noted<br>*Give P values as exact values whenever suitable.*
☒	☐	For Bayesian analysis, information on the choice of priors and Markov chain Monte Carlo settings
☒	☐	For hierarchical and complex designs, identification of the appropriate level for tests and full reporting of outcomes
☒	☐	Estimates of effect sizes (e.g. Cohen's d, Pearson's r), indicating how they were calculated

Our web collection on statistics for biologists contains articles on many of the points above.

Software and code

Policy information about availability of computer code

Data collection	MinKNOW software v22.08.9 (Oxford Nanopore Technology) for long-read sequencing data

Data analysis	R programming language v 4.3.1<br>tidyverse package v1.3.0<br>cooccur package v1.3<br>pheatmap v 1.0.12<br>ggplot v 3.4.3<br>SPAdes v3.11.1<br>Seqkit v0.15.0<br>Abricate v0.0.9<br>Guppy v6.4.2<br>Nanoplot v1.38.1<br>guppy_barcoder from Guppy v6.3.8<br>flye v2.9.1<br>MinKNOW software v22.08.9<br>MLST v2.23.0<br>AMRFinderPlus v3.11.20<br>ggpubr v0.4.0<br>RColorBrewer v1.1-2<br><br>Analysis code is available at: https://github.com/FEGraf/CHL-Malawi and https://doi.org/10.5281/zenodo.13861919

For manuscripts utilizing custom algorithms or software that are central to the research but not yet described in published literature, software must be made available to editors and reviewers. We strongly encourage code deposition in a community repository (e.g. GitHub). See the Nature Portfolio guidelines for submitting code & software for further information.

Data

Policy information about availability of data

All manuscripts must include a data availability statement. This statement should provide the following information, where applicable:

- Accession codes, unique identifiers, or web links for publicly available datasets
- A description of any restrictions on data availability
- For clinical datasets or third party data, please ensure that the statement adheres to our policy

Data Availability
Supplementary Figures 1-9 and supplementary Tables 1 & 2 are accessible in the supplementary information of this manuscript. Reads from all isolates previously sequenced and used in this study are accessible in the European Nucleotide Archive (ENA) under project IDs PRJEB8265, PRJEB26677, PRJEB28522 and PRJEB36486 and ENA accession numbers for isolates linked to metadata are available in the Supplementary Data 1. Long-read sequence data have been submitted to the National Center for Biotechnology Information (NCBI) under the BioProject ID PRJNA1040831, BioSample accession numbers for sequenced isolates are in Supplementary Table 3. Source data are provided with this paper and are also accessible from the GitHub repository https://github.com/FEGraf/CHL-Malawi, https://zenodo.org/records/13861919

Research involving human participants, their data, or biological material

Policy information about studies with human participants or human data. See also policy information about sex, gender (identity/presentation), and sexual orientation and race, ethnicity and racism.

Reporting on sex and gender	NA
Reporting on race, ethnicity, or other socially relevant groupings	NA
Population characteristics	NA
Recruitment	NA
Ethics oversight	NA

Note that full information on the approval of the study protocol must also be provided in the manuscript.

Field-specific reporting

Please select the one below that is the best fit for your research. If you are not sure, read the appropriate sections before making your selection.

☒ Life sciences ☐ Behavioural & social sciences ☐ Ecological, evolutionary & environmental sciences

For a reference copy of the document with all sections, see nature.com/documents/nr-reporting-summary-flat.pdf

Life sciences study design

All studies must disclose on these points even when the disclosure is negative.

Sample size	No statistical method was used to predetermine sample size. We investigated all available genomes from E. coli and Klebsiella available at the time from Blantyre, Malawi.
Data exclusions	No data has been excluded.
Replication	All experimental replicates (where applicable) were successful for phenotypic and functional assays (number of repeats given for each assay).
Randomization	The experiments were not randomized. Randomization was not relevant as this was a detailed investigation of specific bacterial isolates guided by their genotype.
Blinding	The Investigators were not blinded to allocation during experiments and outcome assessment. Blinding was not relevant as this was a detailed investigation of specific bacterial isolates guided by their genotype.

Reporting for specific materials, systems and methods

We require information from authors about some types of materials, experimental systems and methods used in many studies. Here, indicate whether each material, system or method listed is relevant to your study. If you are not sure if a list item applies to your research, read the appropriate section before selecting a response.

Materials & experimental systems

n/a	Involved in the study	
☒	☐	Antibodies
☒	☐	Eukaryotic cell lines
☒	☐	Palaeontology and archaeology
☒	☐	Animals and other organisms
☒	☐	Clinical data
☒	☐	Dual use research of concern
☒	☐	Plants

Methods

n/a	Involved in the study	
☒	☐	ChIP-seq
☒	☐	Flow cytometry
☒	☐	MRI-based neuroimaging

Plants

Seed stocks	NA
Novel plant genotypes	NA
Authentication	NA
